# Identification of a putative Gag binding site critical for feline immunodeficiency virus genomic RNA packaging

**DOI:** 10.1261/rna.079840.123

**Published:** 2024-01

**Authors:** Anjana Krishnan, Lizna M. Ali, Suresha G. Prabhu, Vineeta N. Pillai, Akhil Chameettachal, Valérie Vivet-Boudou, Serena Bernacchi, Farah Mustafa, Roland Marquet, Tahir A. Rizvi

**Affiliations:** 1 Department of Microbiology and Immunology, College of Medicine and Health Sciences (CMHS), United Arab Emirates University (UAEU), Al Ain, United Arab Emirates; 2 Université de Strasbourg, CNRS, Architecture et Réactivité de l'ARN, UPR 9002, 67084 Strasbourg cedex, France; 3 Department of Biochemistry, College of Medicine and Health Sciences (CMHS), United Arab Emirates University (UAEU), Al Ain, United Arab Emirates; 4 Zayed bin Sultan Center for Health Sciences, United Arab Emirates University, Al Ain, United Arab Emirates; 5 ASPIRE Research Institute in Precision Medicine, Abu Dhabi, United Arab Emirates

**Keywords:** retroviruses, feline immunodeficiency virus, RNA packaging signal (ψ), RNA secondary structure, hSHAPE, Gag/RNA interactions, filter-binding assays

## Abstract

The retroviral Gag precursor plays a central role in the selection and packaging of viral genomic RNA (gRNA) by binding to virus-specific packaging signal(s) (psi or ψ). Previously, we mapped the feline immunodeficiency virus (FIV) ψ to two discontinuous regions within the 5′ end of the gRNA that assumes a higher order structure harboring several structural motifs. To better define the region and structural elements important for gRNA packaging, we methodically investigated these FIV ψ sequences using genetic, biochemical, and structure–function relationship approaches. Our mutational analysis revealed that the unpaired U^85^CUG^88^ stretch within FIV ψ is crucial for gRNA encapsidation into nascent virions. High-throughput selective 2′ hydroxyl acylation analyzed by primer extension (hSHAPE) performed on wild type (WT) and mutant FIV ψ sequences, with substitutions in the U^85^CUG^88^ stretch, revealed that these mutations had limited structural impact and maintained nucleotides 80–92 unpaired, as in the WT structure. Since these mutations dramatically affected packaging, our data suggest that the single-stranded U^85^CUG^88^ sequence is important during FIV RNA packaging. Filter-binding assays performed using purified FIV Pr50^Gag^ on WT and mutant U^85^CUG^88^ ψ RNAs led to reduced levels of Pr50^Gag^ binding to mutant U^85^CUG^88^ ψ RNAs, indicating that the U^85^CUG^88^ stretch is crucial for ψ RNA–Pr50^Gag^ interactions. Delineating sequences important for FIV gRNA encapsidation should enhance our understanding of both gRNA packaging and virion assembly, making them potential targets for novel retroviral therapeutic interventions, as well as the development of FIV-based vectors for human gene therapy.

## INTRODUCTION

Feline immunodeficiency virus (FIV) is a lentivirus that causes chronic, often lethal diseases with prolonged incubation periods in different species of domestic and big cats (Kenyon and Lever 2011). Similar to the human immunodeficiency virus type 1 (HIV-1) that causes acquired immunodeficiency syndrome (AIDS) in humans, FIV infection in domestic and wild cats compromises the host immune system, resulting in full-blown feline AIDS (Pedersen et al. 1987; Sparger et al. 1989; Elder 1993; Pedersen 1993; Luttge and Freed 2010; Kenyon and Lever 2011). There has been increasing interest in studying the replication and pathophysiology of FIV in recent years, because FIV/cat is the only available nonprimate model for AIDS in its natural host where anti-AIDS vaccines and therapies can be tested efficiently (Olmsted et al. 1989; Poeschla et al. 1998; Miller et al. 2000; Elder et al. 2008; Kenyon and Lever 2011; Bienzle 2014; Kim et al. 2023). In addition, there has been a push to develop FIV vectors for human gene therapy as an alternative to HIV-1-based vectors. For one, FIV is phylogenetically distant from primates, reducing the chance of recombination with human endogenous and exogenous viruses. Furthermore, being a lentivirus, FIV is able to infect nondividing cells, such as muscles, brain, and cardiac cells, which are the actual targets of human gene therapy (Poeschla et al. 1998; Wang et al. 1999; Price et al. 2002; Saenz and Poeschla 2004; Barraza and Poeschla 2008; Kenyon and Lever 2011; Poeschla 2011). This makes it important to study pertinent features of the FIV replication cycle, especially those involved in the encapsidation of the FIV genomic RNA (gRNA) into the assembling virions.

Genomic RNA packaging occurs concomitantly with viral assembly, resulting in preferential encapsidation of the virus genome (which is ∼1% or less of the total mRNA present in the infected cell) over other spliced viral and host mRNAs that can be found within the host cell (D'Souza and Summers 2005; Lever 2007; Rulli et al. 2007; Johnson and Telesnitsky 2010; Lu et al. 2011a,b; Miyazaki et al. 2011; Kutluay et al. 2014; Ali et al. 2016; Comas-Garcia et al. 2016; Kaddis Maldonado and Parent 2016; Bieniasz and Telesnitsky 2018; Dubois et al. 2018; Chameettachal et al. 2023). It is now well established that gRNA encapsidation is mediated by *cis*-acting sequence(s) present in the 5′ untranslated leader region (5′ UTR) of the viral genome and may extend into the *gag* (Berkhout and van Wamel 1996; Das et al. 1997; Clever et al. 2002; D'Souza and Summers 2005; Mustafa et al. 2005; Lever 2007; Kenyon et al. 2008; Johnson and Telesnitsky 2010; Rizvi et al. 2010; Jouvenet et al. 2011; Ali et al. 2016; Comas-Garcia et al. 2016; Kaddis Maldonado and Parent 2016; Mailler et al. 2016; Dubois et al. 2018; Chameettachal et al. 2023). Referred to as psi (ψ), these sequences are critical in recognizing and capturing the Gag precursor polyprotein to initiate the process of encapsidation (D'Souza and Summers 2005; Lever 2007; Johnson and Telesnitsky 2010; Ali et al. 2016; Comas-Garcia et al. 2016; Kaddis Maldonado and Parent 2016; Dubois et al. 2018; Chameettachal et al. 2023). Acting at the secondary structure level, these sequences assume higher order structural motifs found to be important for gRNA packaging among various retroviruses (Clever et al. 1995; Zeffman et al. 2000; D'Souza and Summers 2004, 2005; Mustafa et al. 2004, 2018; Lever 2007; Kenyon et al. 2008, 2011; Jaballah et al. 2010; Johnson and Telesnitsky 2010; Rizvi et al. 2010; Aktar et al. 2013, 2014; Ali et al. 2016; Comas-Garcia et al. 2016; Kaddis Maldonado and Parent 2016; Dubois et al. 2018; Chameettachal et al. 2023). Interestingly, it is the existence of these structural motifs that explains the phenomenon of cross-/copackaging observed among diverse retroviruses in the absence of any sequence homology (Embretson and Temin 1987; Rizvi and Panganiban 1993; Yang and Temin 1994; Das et al. 1997; Yin and Hu 1997; Certo et al. 1998; White et al. 1999; Browning et al. 2001; Al Dhaheri et al. 2009; Al Shamsi et al. 2011; Ali et al. 2016; Chameettachal et al. 2023). However, specific sequences in the context of the structural motifs within ψ are important to allow the initial interaction of the gRNA with the Gag precursor protein, as has recently been shown for HIV-1, Mason–Pfizer monkey virus (MPMV), and mouse mammary tumor virus (MMTV) (Abd El-Wahab et al. 2014; Chameettachal et al. 2021; Pitchai et al. 2021).

The FIV packaging determinants are complex with sequences present both upstream and downstream from the major splice donor (mSD) at the 5′ end of the FIV genome (Kemler et al. 2002, 2004, Browning et al. 2003a,b). These determinants were observed to be bipartite in nature, with the first region spanning from +1 in R to 291 nt within the 5′ UTR, and the second region residing within the first 100 nt of *gag*, with the intervening 120 nt within the UTR being dispensable for packaging (Mustafa et al. 2005). In addition, sequences within the 3′ FIV long terminal repeat (LTR) were also observed to affect RNA packaging, though marginally (Ghazawi et al. 2006). The core packaging determinants were predicted to fold into several stem–loops and maintain long-range interactions (LRIs) between a heptanucleotide in R/U5 and Gag coding sequences (Kenyon et al. 2008). This predicted higher-order structure was supported by conventional biochemical mapping, phylogenetic analysis, and biological assays, which also confirmed the existence of the R/U5 and Gag LRI (Kenyon et al. 2008; Rizvi et al. 2010). However, in contrast, Sargueil and colleagues proposed different higher-order structures using phylogeny as well as chemical and enzymatic probing (James and Sargueil 2008). A later study resolved the discrepancy using high-throughput selective 2′ hydroxyl acylation analyzed by primer extension (hSHAPE) of the FIV ψ, further validating the existence of the R/U5 and Gag LRI, but at the same time revealing that the 5′ and 3′ sequences involved in forming the LRI can also assume an alternative conformation by occluding the dimerization initiation site (DIS). Hence, it has been proposed that this alternative conformation potentially favors translation and splicing over encapsidation (Kenyon et al. 2011).

Despite being investigated quite thoroughly, it remains largely unclear which particular sequence(s) within the FIV ψ plays an important role during FIV gRNA packaging via specific recognition by the Gag precursor, as has recently been demonstrated for HIV-1, MMTV, MPMV, and MoMuLV (D'Souza and Summers 2004; Gherghe et al. 2010; Abd El-Wahab et al. 2014; Chameettachal et al. 2021; Ding et al. 2021; Pitchai et al. 2021). Therefore, we systematically introduced mutations in different parts of FIV ψ sequences toward establishing the biological significance of a particular sequence(s) and/or of a structural component(s) during FIV RNA packaging. These mutants were then tested using a combination of in-cell genetic complementation, biochemical, and structure–function relationship approaches. To further understand how the Gag structural polyprotein (Pr50^Gag^) captures FIV gRNA, we used the recently purified full-length FIV Gag protein (Krishnan et al. 2019) to study its interaction with the FIV ψ sequences. Our results demonstrate that a stretch of unpaired U^85^CUG^88^ in the FIV ψ is crucial for FIV gRNA packaging and constitutes a potential Gag binding site.

## RESULTS

### Purine-rich regions in the vicinity of the DIS do not play a role in the packaging and propagation of FIV gRNA

**FIGURE 1.**
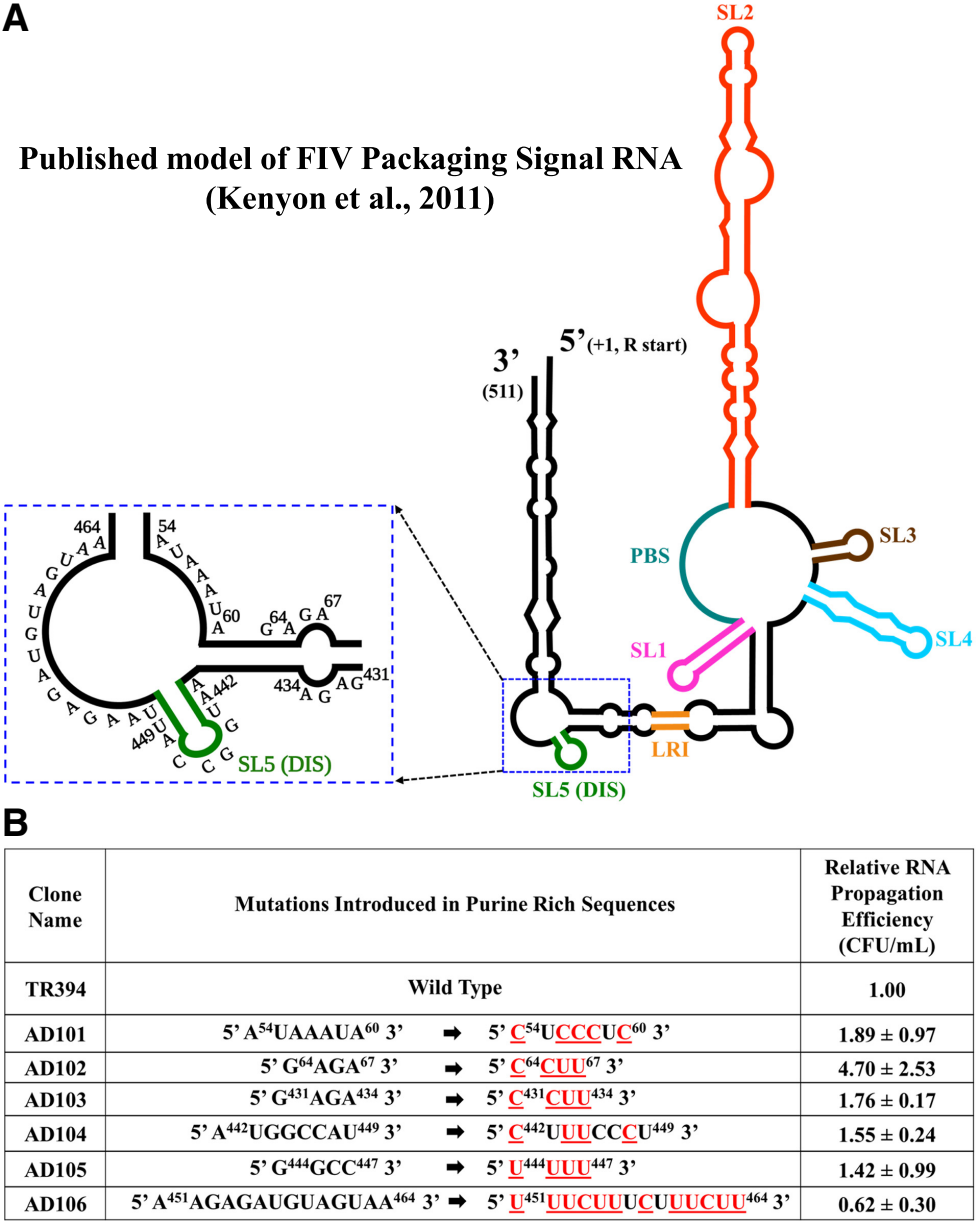
Test of purine-rich sequences in the vicinity of DIS during FIV gRNA packaging and propagation. (*A*) Schematic drawing of the previously published secondary structure of FIV packaging signal RNA (Kenyon et al. 2011). For clarity, the purine-rich region targeted for introducing substitution mutations is enlarged and shown as an *inset*. (*B*) Description of mutations introduced in the selected purine-rich sequences. Red and underlined nucleotides represent the substitutions introduced. The nucleotide positions are shown as superscripts. The relative RNA propagation efficiency of each mutant RNA is shown in the *far-right* column as hygromycin-resistant (Hyg^R^) colony-forming units per mL (CFU/mL) normalized to the transfection efficiency. The data shown here are from a minimum of three independent experiments performed in triplicate (± standard deviation [SD]).

Several recent studies have suggested that unpaired purines or purine-rich sequences play an important role in retroviral gRNA packaging (Damgaard et al. 1998; Baig et al. 2009; Jaballah et al. 2010; Abd El-Wahab et al. 2014; Bernacchi et al. 2017; Mustafa et al. 2018; Wu et al. 2018; Ali et al. 2020; Nikolaitchik et al. 2020; Chameettachal et al. 2021; Pillai et al. 2021; Pitchai et al. 2021; Umunnakwe et al. 2021). Therefore, to understand the role of purines in the packaging of FIV gRNA, we introduced substitution mutations in several stretches of purine-rich regions of the FIV packaging signal RNA whose secondary structure was previously published (Kenyon et al. 2011). First, we introduced substitution mutations in the purine-rich regions found in close proximity to the previously reported DIS of FIV gRNA (Fig. 1; Kenyon et al. 2008, 2011; Rizvi et al. 2010). We were particularly interested in investigating this region since it has been shown for other retroviruses that sequences overlapping the DIS (Lanchy et al. 2003; Umunnakwe et al. 2021), or positioned in close proximity of the DIS, facilitate gRNA packaging by acting as potential Gag binding sites (Abd El-Wahab et al. 2014; Smyth et al. 2015; Bernacchi et al. 2017; Chameettachal et al. 2021; Pillai et al. 2021; Pitchai et al. 2021).

Substitution mutations were introduced in the FIV packaging signal RNA that replaced the purines with pyrimidines (AD101–AD106; Fig. 1A,B). The effects of these mutations on gRNA packaging and propagation efficiency were tested using our previously established single round of replication assay (Supplemental Fig. S1; Browning et al. 2001). Surprisingly, none of the tested mutants showed any significant adverse effects on gRNA propagation compared to wild type (WT), suggesting that the mutated purine-rich regions were not essential for FIV gRNA packaging (Fig. 1B). In fact, mutations in AD104 and AD105, which destroyed the palindromicity of the previously described DIS (Kenyon et al. 2008, 2011), showed a 1.5-fold increase in RNA propagation (Fig. 1B). This suggests that the initially proposed DIS may not be the actual DIS and there may exist other sequences in the packaging signal RNA which may act as the DIS. Since none of our mutants showed significant reduction in RNA propagation, we did not investigate their relative packaging efficiencies. Indeed, RNA propagation is a post-RNA packaging phenomenon which in our assay is dependent upon successful RNA packaging, reverse transcription, and integration of the reverse transcribed DNA.

### Stem–loop 1 does not directly affect the packaging and propagation of FIV gRNA

**FIGURE 2.**
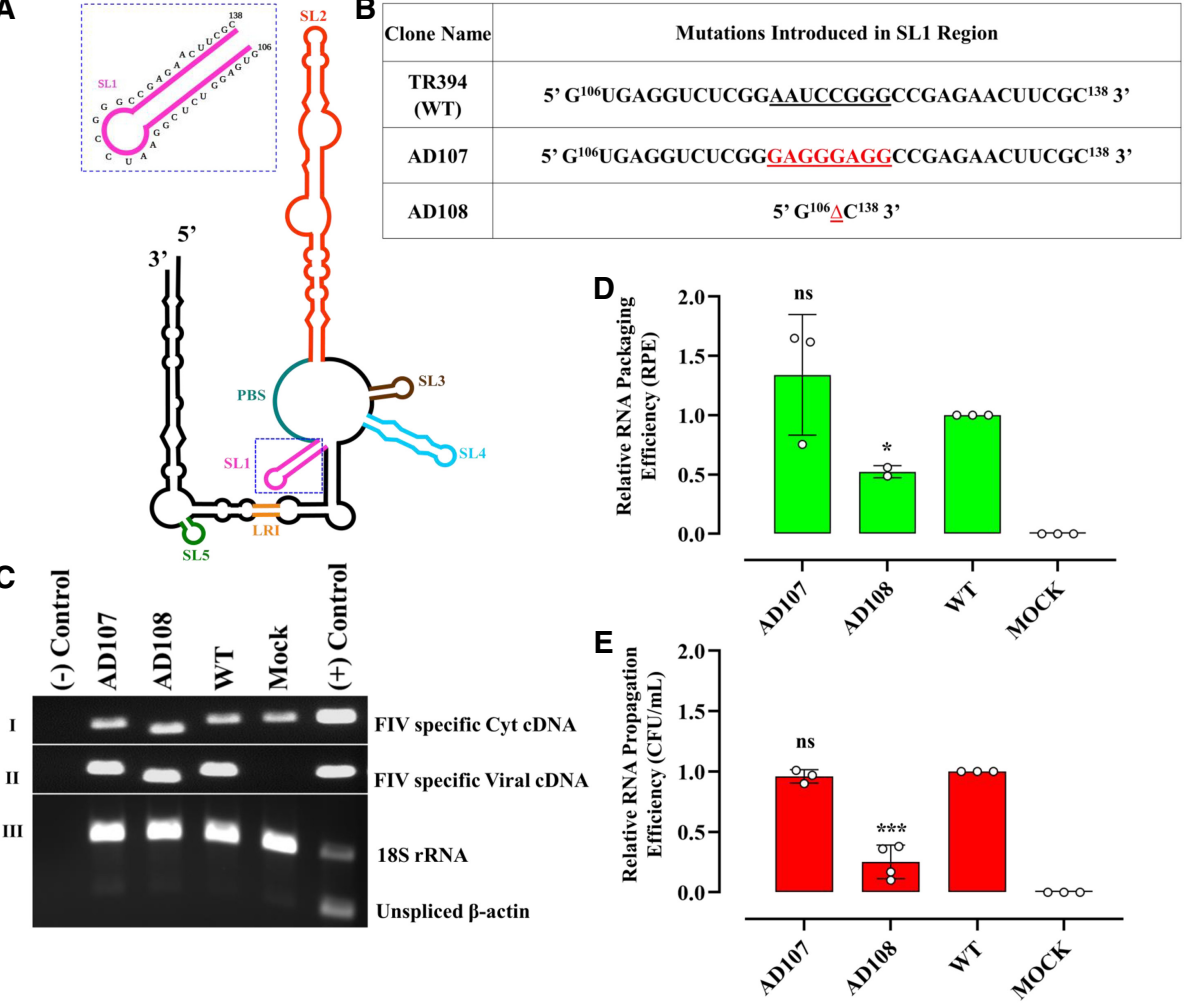
Role of SL1 in FIV gRNA packaging and propagation. (*A*) Schematic drawing of the previously published secondary structure of FIV packaging signal RNA (Kenyon et al. 2011). The SL1 region with its nucleotide sequences is enlarged and shown as an *inset*. (*B*) Description of mutations introduced in SL1. (*C*) Gel images showing polymerase chain reaction (PCR) amplification of cytoplasmic and viral cDNA with appropriate controls. Panels I and II: PCR amplifications using FIV-specific primers (210 bp). Panel III: Multiplex PCR amplifications performed on cytoplasmic cDNAs using primers/competimer for 18S rRNA (324 bp) and unspliced β-actin mRNA (200 bp). (*D*) Relative RNA packaging efficiency (RPE) of the mutant transfer vector RNAs using quantitative RT-PCR. (*E*) The relative RNA propagation efficiency of the mutant RNAs is given as Hyg^R^ CFU/mL normalized to the transfection efficiency. The data shown in the histograms are from a minimum of three independent experiments (shown as open circles) performed in triplicate (±SD) except for the RPE of AD108 where two values were used. Mock transfection cocktail contained only WT transfer vector (TR394) and no packaging construct. Statistical significance: ns, not significant; (*) 0.01 ≤ *P* < 0.05, (***) 0.0001 ≤ *P* < 0.001.

As mentioned earlier, the FIV RNA packaging determinants are comprised of two disjointed regions (one harbors sequences from +1 in R to 291 nt within the 5′ UTR and the second contains the first 100 nt of Gag [Mustafa et al. 2005]). Interestingly, the intervening sequences shown to be dispensable for FIV RNA packaging are involved in forming two stem–loops (SLs), SL3, and SL4 (Kenyon et al. 2011). Therefore, we investigated SL1 located in close proximity of the primer binding site (PBS) (Fig. 2A), whose role has not yet been elucidated in FIV gRNA packaging. Two mutations were introduced in SL1: (i) a substitution of the 8-nt loop with two tandem repeats of the tetrad GAGG (AD 107; Fig. 2B) and (ii) a complete deletion of SL1 (AD108; Fig. 2B).

To ensure that the introduced mutations did not adversely affect the transfer vector RNA stability and expression, which could have compromised its ability to get packaged into the budding virions, we analyzed both the cytoplasmic as well as viral RNA preparations using RT-PCR (see Materials and Methods). PCR amplification of the cDNA confirmed cDNA quality (Fig. 2C; panels I, II, respectively). We also validated the fractionation technique that we used to ensure that the transfer vector RNAs were properly exported out of the nucleus by performing RT-PCR of unspliced β-actin mRNA (Fig. 2C, panel III) since it should remain in the nucleus (Tan et al. 1995). The presence of bands for 18S ribosomal RNA (rRNA) (Fig. 2C; panel III) in the multiplex PCR confirmed the presence, quality, and amplifiability of cDNAs in the same reaction mix.

Replacing the 8-nt loop of SL1 with two GAGG tandem repeats did not show any defect either at the RNA packaging or RNA propagation levels (mutant AD107 in Fig. 2D,E, respectively). This suggested that the 8-nt loop of SL1 is not involved in FIV gRNA packaging. On the other hand, deletion of the entire SL1 (AD108) resulted in (∼60%) reduction in RNA packaging compared to the WT that was statistically significant (*P*-value = 0.04) (Fig. 2D). Since the SL1 mutation is rather large and SL1 is part of a large central domain holding SL1–SL4, this effect could have been due to an overall effect on the 3D RNA structure. Analogous to RNA packaging, a similar but distinct reduction in RNA propagation was also observed in this mutant (*P*-value = 0.001) (Fig. 2E). The more pronounced defect in RNA propagation of this mutant could be due to the proximity of SL1 to the PBS that could have affected reverse transcription (Mikkelsen et al. 1996; Oh et al. 2008; Aktar et al. 2014; Chameettachal et al. 2021). Taken together, these results suggest that sequences within SL1 are not directly involved in FIV gRNA packaging.

### A UCUG stretch plays a crucial role in FIV gRNA packaging

**FIGURE 3.**
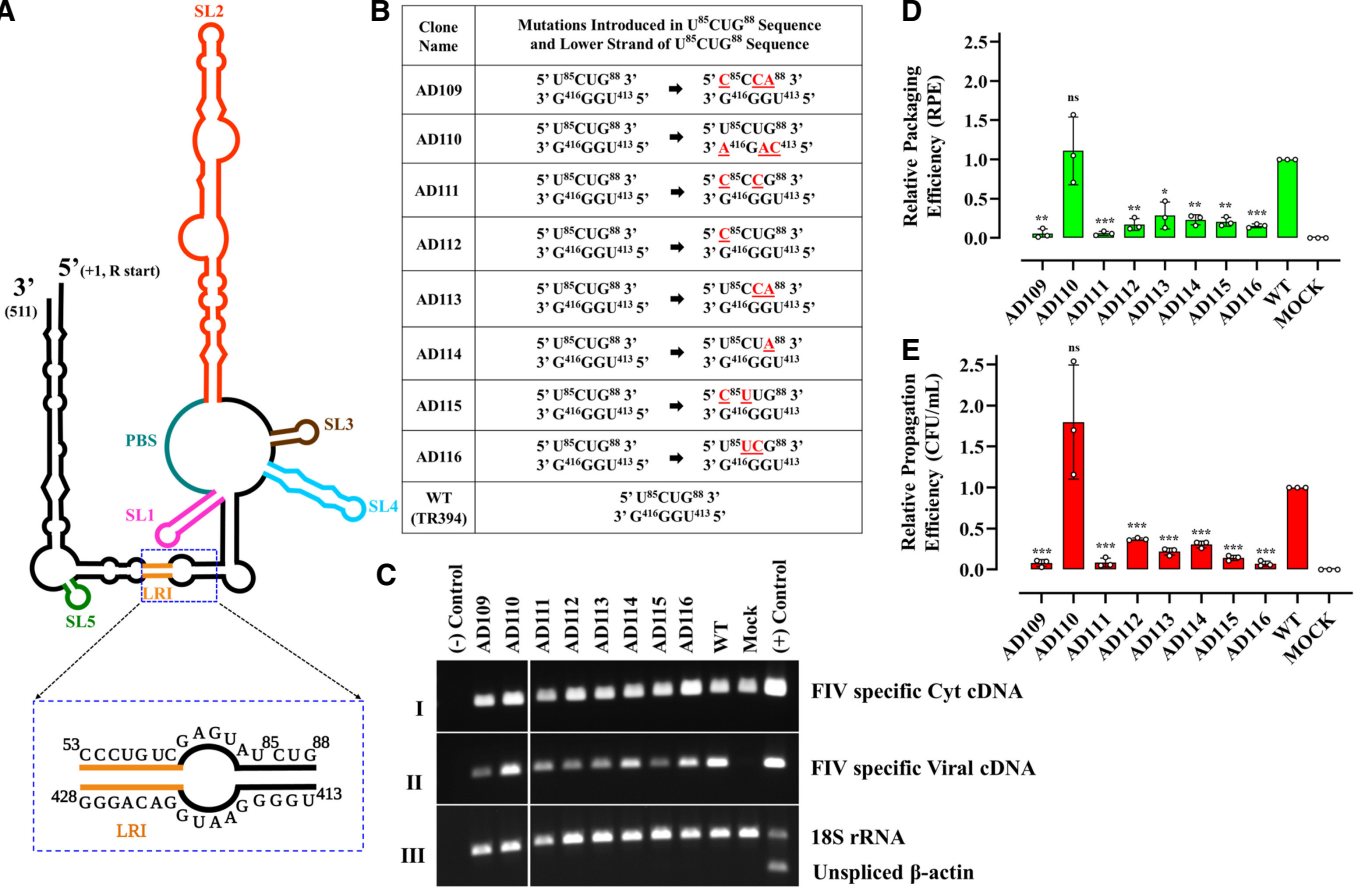
The U^85^CUG^88^ stretch plays a crucial role in FIV gRNA packaging. (*A*) Schematic drawing of the previously published secondary structure of FIV packaging signal RNA (Kenyon et al. 2011). Position of LRI, U^85^CUG^88^ and its complementary strand sequences are enlarged and shown as an *inset*. (*B*) Description of mutations introduced in U^85^CUG^88^ and its complementary strand. (*C*) Gel images showing PCR amplification of cytoplasmic and viral cDNAs with appropriate controls. PCR amplifications using FIV-specific primers (210 bp) on panel I: cyt and panel II: viral cDNAs. Panel III: Multiplex PCR amplifications performed on cytoplasmic cDNAs using primers/competimer for 18S rRNA (324 bp) and unspliced β-actin mRNA (200 bp). (*D*) Relative RPE of the mutant transfer vector RNAs using quantitative real-time RT-PCR. (*E*) The relative RNA propagation efficiencies of the mutant RNAs are shown as Hyg^R^ CFU/mL normalized to the transfection efficiency. The data shown in the histograms are from a minimum of three independent experiments (shown as open circles) performed in triplicate (±SD). Mock transfection cocktail contained only WT transfer vector (TR394) and no packaging construct. Statistical significance: ns, not significant; (*) 0.01 ≤ *P* < 0.05, (**) 0.001 ≤ *P* < 0.01, (***) 0.0001 ≤ *P* < 0.001.

Next, a careful examination of the SHAPE-validated FIV packaging signal RNA revealed two UCUG stretches, both of which are present in base-paired conformation. The first one is present at nucleotides 85–88 (U^85^CUG^88^), immediately downstream from the LRI, while the other one is located at nucleotides 332–335, which is at the base of SL3 (Fig. 3; Kenyon et al. 2008). However, the sequences involved in forming SL3 and SL4 have been shown to be dispensable for FIV RNA packaging and propagation (Mustafa et al. 2005), indicating that the second UCUG motif at nucleotides 332–335 could not be considered important during FIV gRNA packaging. Therefore, the U^85^CUG^88^ stretch was interrogated, especially keeping in mind earlier studies that have shown that following dimerization, Moloney murine leukemia virus (MoMuLV) packaging signal RNA undergoes a conformational change exposing a conserved UCUG motif in a loop (D'Souza and Summers 2004; Gherghe et al. 2010; Miyazaki et al. 2010). In MoMuLV, these UCUG stretches are base paired in the monomeric gRNA and have the tendency to bind to MoMuLV Gag as well as nucleocapsid (NC) proteins with high affinity in vitro (D'Souza and Summers 2004; Gherghe et al. 2010; Miyazaki et al. 2010). Interestingly enough, in the SHAPE structure proposed by Kenyon et al. (2011), the U^85^CUG^88^ stretch in the FIV packaging signal is base paired with U^413^GGG^416^, thus forming a single Watson–Crick base pair and three Wobble base pairs. The stabilization of this base pairing by introducing substitution mutations that converted the Wobble base pairs to Watson–Crick base pairs (U^85^CUG^88^ → C^85^CCA^88^) allowed Kenyon et al. (2011) to conclude that, according to their biochemical studies, the FIV packaging signal RNA adopts an LRI conformation that would favor packaging.

In order to study the effect of mutations in the U^85^CUG^88^ stretch and in its base paired sequence (U^413^GGG^416^) on FIV gRNA packaging and propagation, we introduced the same mutation (U^85^CUG^88^ → C^85^CCA^88^) that was tested by Kenyon and colleagues for in vitro biochemical experiments. This mutation was cloned in the FIV transfer vector (AD109; Fig. 3A,B) for testing its effect on RNA packaging and propagation in a biologically relevant genetic complementation assay. In an alternative approach, we also introduced mutations in the U^413^GGG^416^ sequence in a fashion that converted the Wobble base pairs into the canonical Watson–Crick base pairs (U^413^GGG^416^ → C^413^AGA^416^; AD110) (Fig. 3A,B). After confirming the proper nucleocytoplasmic fractionation, stability, and nuclear export of mutant transfer vector RNAs (Fig. 3C), we quantified the levels of cytoplasmic transfer vector RNA expression and the amount of RNA packaged into the virus particles. Contrary to our expectations, substitutions in the “upper” strand (U^85^CUG^88^ → C^85^CCA^88^) that are expected to stabilize base pairing through Watson–Crick interactions showed a drastic reduction in both gRNA packaging and propagation (RPE = 0.02 and CFU/mL = 0.06, respectively; mutant AD109 in Fig. 3D,E). On the other hand, substitutions in the “lower” strand (also predicted to stabilize the base pairing interactions) did not exert any adverse effect on gRNA packaging and propagation (Fig. 3D,E, mutant AD110). These results suggest that the substitutions (U^85^CUG^88^ → C^85^CCA^88^) in the upper strand could not be tolerated by the virus, emphasizing the importance of the U^85^CUG^88^ sequence in FIV gRNA packaging. On the other hand, substitutions in the lower strand could be tolerated very well, suggesting that these sequences do not play an important role in FIV gRNA packaging. This observation further argues that the U^85^CUG^88^ stretch may not be in a base paired state, as has been initially proposed, an observation that was based solely on in vitro biochemical approaches (Kenyon et al. 2011).

Having established that the U^85^CUG^88^ stretch is indispensable for FIV RNA packaging and could potentially serve as the Gag binding site, a series of targeted substitution mutations were introduced within this stretch (mutants AD111–116; Fig. 3B) in order to determine which particular nucleotide(s) could be crucial in FIV gRNA packaging. All the targeted substitutions in any of the 4 nt within the U^85^CUG^88^ motif in different combinations resulted in drastic defects in both RNA packaging and vector RNA propagation in a highly statistically significant manner (Fig. 3D,E). These results clearly show that the 4 nt in the U^85^CUG^88^ stretch function synergistically and are critical determinants of efficient RNA packaging and propagation, perhaps by serving as the initial site for Gag interaction, as has been shown previously for MoMuLV (D'Souza and Summers 2004; Gherghe et al. 2010; Miyazaki et al. 2010).

### All U^85^CUG^88^ mutant RNAs dimerize efficiently

**FIGURE 4.**
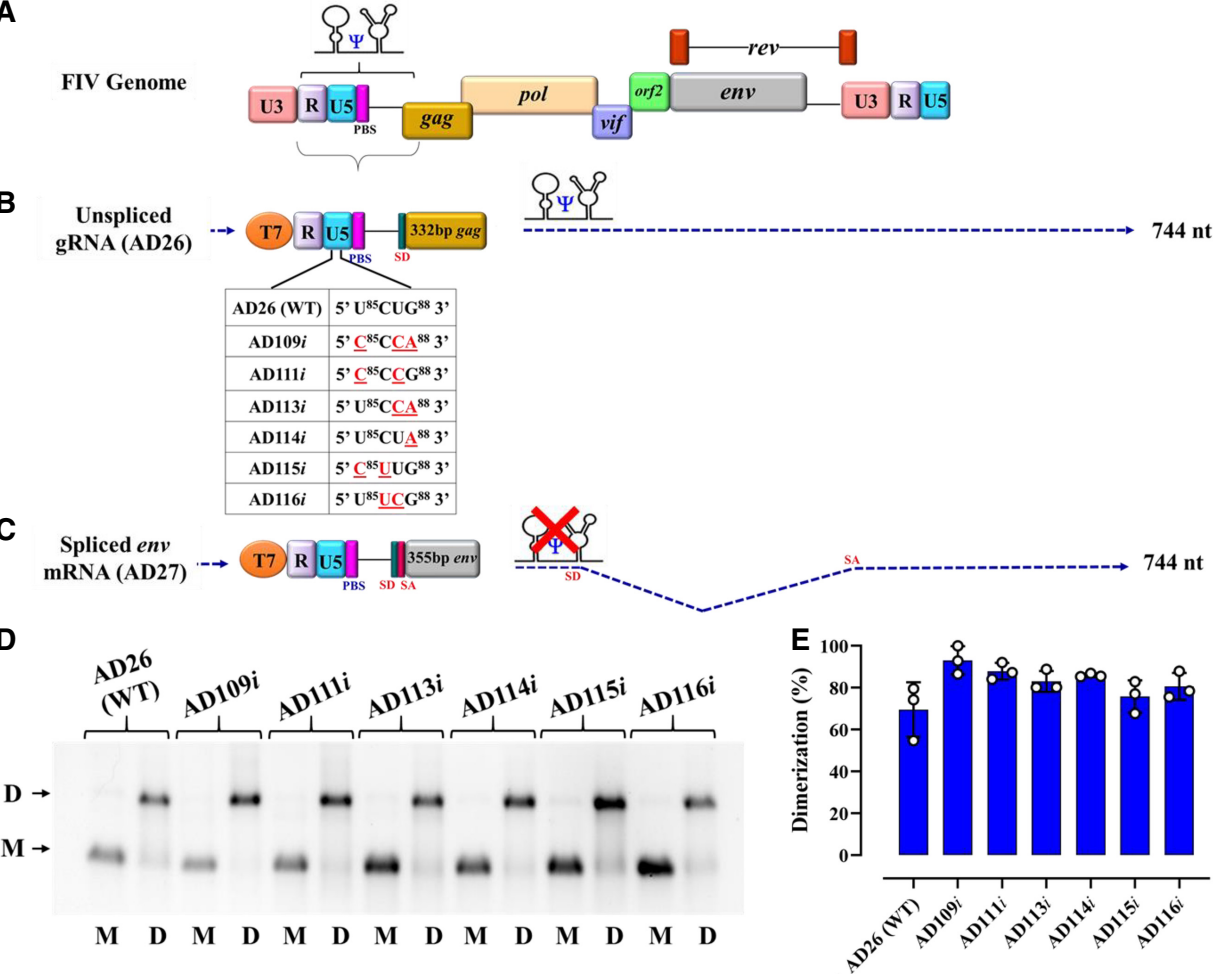
Dimerization of the U^85^CUG^88^ mutants is not affected by the introduced mutations. Schematic representation of: (*A*) The FIV genome, (*B*) the full-length unspliced gRNA up to 744 nt expressed from a T7 expression plasmid. The dashed arrow shows the 744-nt long, 5′ region of the unspliced FIV gRNA that was cloned for in vitro transcription. The table describes the different mutations introduced in the U^85^CUG^88^ motif of the gRNA for biochemical experiments. (*C*) The *envelope (env)* spliced mRNA up to 744 nt expressed from a T7 expression plasmid. The dashed arrow shows the 5′ 744-nt long region of spliced *env* mRNA that was cloned for in vitro transcription. (*D*) A representative gel image showing in vitro dimerization of WT gRNA (AD26) and U^85^CUG^88^ mutant RNAs in TBM buffer. M and D *below* the lanes of the gel indicate the monomer and dimer buffers, respectively, in which the dimerization experiments were performed. The positions of the monomeric and dimeric RNA species are indicated by the letters M and D, respectively, on the *left* side of the gel. (WT) Wild type. (*E*) The histograms show dimerization efficiencies of all mutant RNAs compared to the WT calculated by performing a densitometric analysis of the bands from three independent experiments (shown as open circles). Dimerization efficiency was calculated by measuring the intensity of the dimeric RNA band divided by the intensity of total RNA bands (i.e., sum of intensities of dimer and monomer bands).

Since retroviral RNA dimerization and RNA packaging are highly interlinked processes (D'Souza and Summers 2004; Moore and Hu 2009; Johnson and Telesnitsky 2010; Miyazaki et al. 2011; Aktar et al. 2014; Ali et al. 2016; Dubois et al. 2018; Chameettachal et al. 2023), we analyzed the dimerization capabilities of the U^85^CUG^88^ substitution mutants that showed drastic effects on RNA packaging. Toward this end, we in vitro transcribed the first 744 nt (starting from +1R up to 332 nt into *gag*) of the unspliced WT (AD26) and U^85^CUG^88^ mutant RNAs bearing the same substitution mutations as shown in Figure 3B, creating AD109*i*, AD111*i*, AD113*i*, AD114*i*, AD115*i*, and AD116*i*; Figure 4. As shown in Figure 4D,E, all U^85^CUG^88^ mutants maintained their ability to dimerize similar to the WT RNA, confirming that the reduced RNA packaging and propagation of these mutants was not due to defects in dimerization. These results further directly implicate nucleotides U^85^CUG^88^ in FIV gRNA packaging.

### hSHAPE analyses of FIV packaging signal RNA

The detailed mutational analysis of the U^85^CUG^88^ and U^413^GGG^416^ motifs described above suggested that these regions may indeed not be base paired with each other, as proposed in an earlier secondary structure model (Kenyon et al. 2011). To address this point, we used hSHAPE (McGinnis et al. 2009; Low and Weeks 2010; Weeks and Mauger 2011; Mailler et al. 2019) to interrogate the WT RNA secondary structure with single nucleotide resolution. To achieve this aim, in vitro transcribed RNAs corresponding to the first 744 nt of WT FIV gRNA (AD26, Fig. 4B) were treated with benzoyl cyanide (BzCN), which selectively modifies the RNA by adding acyl groups to single-stranded or flexible regions of the RNA at its 2′-OH ribose groups. The resulting 2′-O-adducts can be detected as stops during the extension of fluorescently labeled primers by reverse transcriptase and analyzed by capillary electrophoresis. The numerical data for the reactivity of each nucleotide obtained from three independent experiments using an updated version of QuShape (Karabiber et al. 2013) was used as pseudoenergy constraints to obtain RNA secondary structure models of the WT FIV packaging sequences with the RNAstructure program (Reuter and Mathews 2010).

**FIGURE 5.**
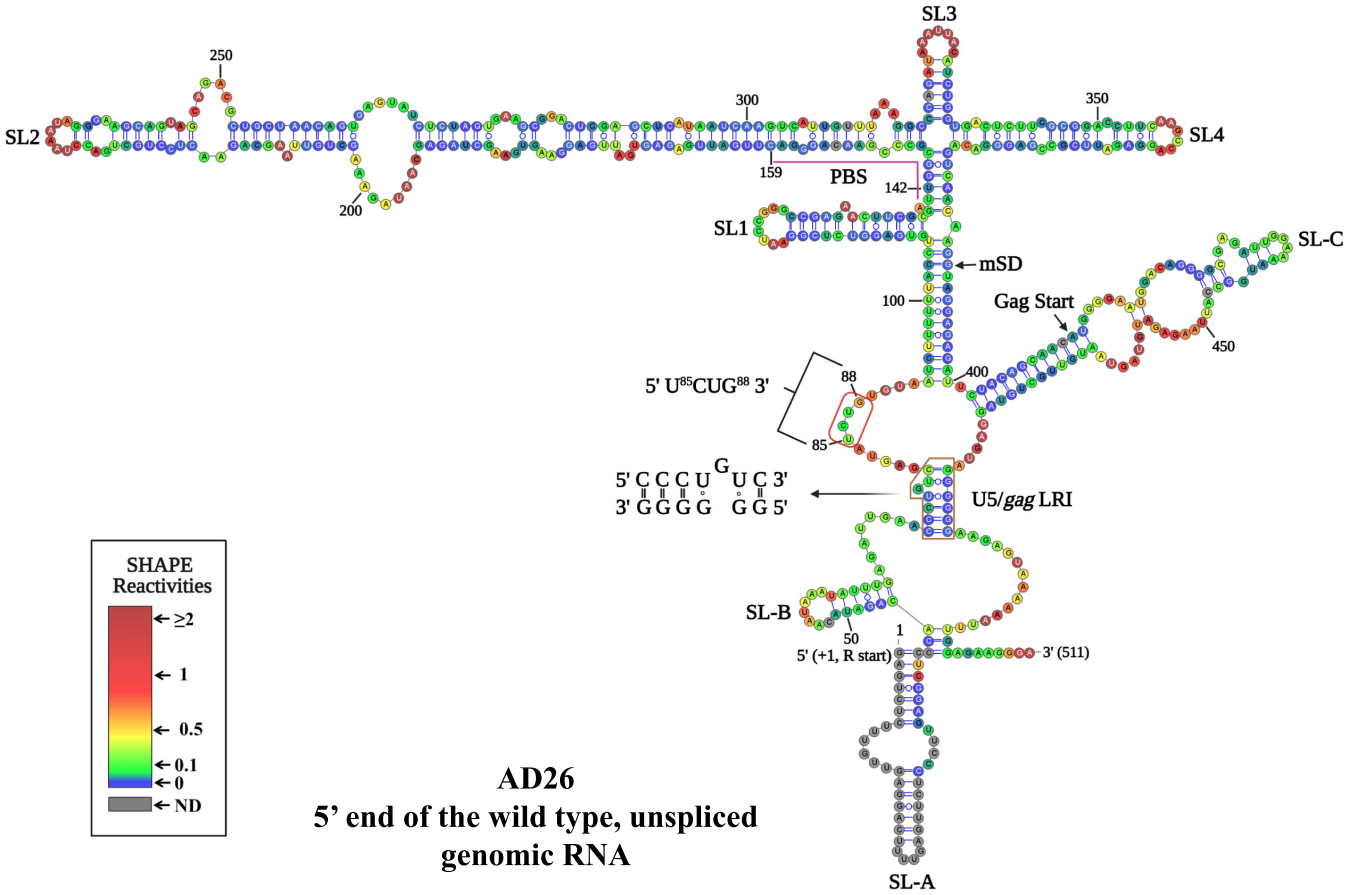
hSHAPE-validated secondary structure model of the first 744 nt of the FIV unspliced gRNA. The in vitro transcribed gRNA was modified with BzCN, and the reactivity of each nucleotide in the RNA secondary structure obtained is color-coded, as indicated in the SHAPE reactivity key. The U5/*gag* LRI is boxed in gold color, and the base pairing of the complementary strands is highlighted and shown as an *inset*. The red box marks the U^85^CUG^88^ motif. The hSHAPE reactivity data are provided in Supplemental Table S2. (PBS) Primer binding site.

As shown in Figure 5, our structural model is in support of the existence of SL1, and a large, stable domain encompassing SL2, SL3, and SL4, which corroborates the model proposed by Kenyon et al. (2011), but with important differences. For instance, three additional SLs, two at the 5′ end (SL-A and B) and one closer to the 3′ end (SL-C), were observed in the structure presented here (Fig. 5). The differences observed in our structure toward the 3′ region (from nucleotides 466–511) when compared to the previously published structure could possibly be due to the lack of reactivity data in this region for the structure proposed by Kenyon et al. (2011). However, the most conspicuous difference between the two models was the U^85^CUG^88^ stretch. In the previously proposed structural model, this motif was base paired with U^413^GGG^416^, whereas in our structural model, the U^85^CUG^88^ motif is single-stranded and part of a rather large internal loop (Fig. 5).

Surprisingly, the central part of this internal loop, including 3 nt of the U^85^CUG^88^ stretch, was weakly reactive (Fig. 5). Our biological data that showed that altering nucleotides U^413^GGG^416^ did not impair FIV RNA packaging or RNA propagation is consistent with the fact that this region is not base paired with U^85^CUG^88^, as initially proposed (Fig. 3). However, this does not preclude the possibility that the U^85^CUG^88^ may interact with another region of the RNA. No alternative interaction was proposed by RNAstructure or Kinefold web server that is able to predict pseudoknots. So, the U^85^CUG^88^ stretch might be involved in a yet-to-be-identified noncanonical tertiary interaction with another part of the RNA.

Another distinguishing feature between the two models involves the LRI. The 5′ strand of the R/U5 LRI (C^73^CCUGUC^79^) was the same in the structural model earlier proposed (Kenyon et al. 2011) and in the present study, but found to be base paired with G^481^GGGGG^486^ (3′ strand in *gag*) in the structural model presented here (Fig. 5) instead of G^422^ACAGGG^428^, as previously proposed (Kenyon et al. 2011). We could establish the importance of the newly proposed LRI by a genetic approach: when the sequences of 5′ and 3′ strands were flipped in a fashion that maintained the complementarity between the two strands along with the same number of Watson–Crick and Wobble base pairing as in the WT, RNA packaging and propagation were retained, suggesting the existence of the proposed LRI during FIV gRNA packaging (Supplemental Fig. S2). In addition, it is important to note that in our structural model, the PBS is base paired as these nucleotides showed weak or reduced SHAPE reactivity (Fig. 5). Similarly, the nucleotides comprising Pal/DIS structure proposed in the earlier structure were found to be base paired in our structural model as they depicted only weak to moderate SHAPE reactivities (Fig. 5).

### Gag preferentially binds to unspliced gRNA

Having identified the nucleotides which are crucial for FIV gRNA packaging, we investigated the ability of FIV packaging signal RNA to bind to purified FIV Gag polyprotein (Pr50^Gag^) (Krishnan et al. 2019). The purified protein was able to assemble in vitro into virus-like particles (VLPs), indicating that it preserved its functional ability to oligomerize/multimerize (Krishnan et al. 2019). To examine the purity and integrity of the purified FIV Pr50^Gag^ protein for RNA–protein interaction studies, SDS–PAGE and western blot analysis were performed. There was no degradation observed in the Pr50^Gag^ protein and a single distinct band of molecular weight 50 kDa was detected on Coomassie brilliant blue staining (Supplemental Fig. S3A), as well as western blotting using α-His_6_ and FIV α-p24 monoclonal antibodies (Supplemental Fig. S3B). Furthermore, the purified Pr50^Gag^ protein was characterized by dynamic light scattering (DLS) to ensure it did not contain any aggregates. In solution, the estimated mean hydrodynamic radius (Rh) based on the volume and number distribution was observed to be ∼3.76 nm corresponding to Pr50^Gag^ dimers under our experimental conditions (Supplemental Fig. S3C,D).

**FIGURE 6.**
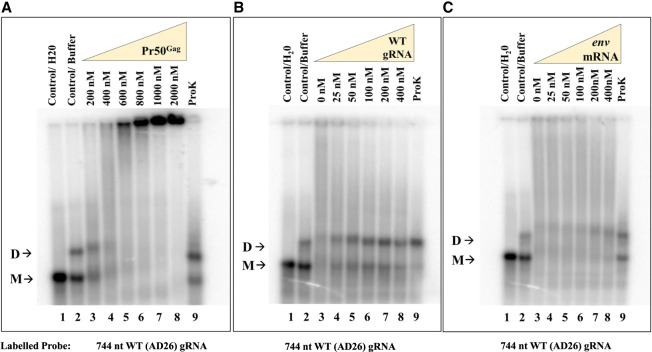
Pr50^Gag^ specifically binds the FIV packaging signal RNA. (*A*) A representative gel of a band-shift assay performed using radiolabeled FIV gRNA (AD26) in the presence of increasing concentration of Pr50^Gag^ showing the formation of Pr50^Gag^-radiolabeled gRNA complexes. (*B*,*C*) Band-shift competition assays showing the differential ability of unspliced WT gRNA (AD26) and spliced *env* mRNA (AD27) to displace Gag binding to the WT labeled gRNA (AD26). Briefly, a 744-nt long, radiolabeled, unspliced WT gRNA (AD26), and 400 nM FIV Pr50^Gag^ were incubated with increasing concentrations of unlabeled unspliced WT gRNA (AD26) or spliced *env* mRNA (AD27) as a competitor. The last lane shows the Proteinase K (ProK)-treated reaction mixture containing unspliced WT gRNA, 400 nM Pr50^Gag^, and 400 nM competitor RNA. M and D denote the monomeric and dimeric forms of RNA, respectively.

In order to test whether Pr50^Gag^ preferentially binds to unspliced gRNA over the viral spliced *env* mRNA, we performed band-shift competition assays using radiolabeled RNA corresponding to the first 744 nt of unspliced gRNA (Fig. 4B) and the same unlabeled RNA or unlabeled spliced *env* mRNA (corresponding to the first 744 nt of the authentic *env* mRNA, Fig. 4C) as competitors. Prior to the band-shift competition assay, a band-shift assay was performed using radiolabeled gRNA and increasing concentrations of Pr50^Gag^ to identify the appropriate concentration of Gag that forms a complex with most or a majority of RNA, thus causing a full-shift at the position of dimeric RNA (Fig. 6A). We observed the formation of RNA–protein complexes and a near complete shift of RNA dimeric band at 400 nM of Pr50^Gag^ (Fig. 6A). Thus, the band-shift competition assays were performed in the presence of 400 nM of Pr50^Gag^. When the protein concentration was increased further, most of the RNA/Pr50^Gag^ complexes remained in the wells (Fig. 6A). In contrast with our previous observations with HIV-1, MMTV, and MPMV Gag proteins (Abd El-Wahab et al. 2014; Chameettachal et al. 2021; Pitchai et al. 2021), the FIV gRNA/Pr50^Gag^ complexes did not form discrete bands during electrophoresis, but instead appear as a continuous smear (Fig. 6A). This indicates that the FIV gRNA/Pr50^Gag^ complexes dissociated during electrophoresis, and thus suggests that binding of FIV Gag protein to its packaging signals is more dynamic than binding of the HIV-1, MMTV, and MPMV Gag to their cognate ψ regions.

Next, we performed band-shift competition assays to test if Pr50^Gag^ preferentially binds to unspliced gRNA over spliced *env* mRNA. In this assay, unlabeled gRNA or *env* mRNA was added to the labeled gRNA as competitor at increasing concentrations and allowed to form protein–RNA complex by adding 400 nM of Pr50^Gag^. In the absence of unlabeled gRNA competitor, all the labeled RNA could bind to Gag, resulting in a complete band-shift (Fig. 6B,C; lane 3). However, adding 25 nM or a higher concentration of unlabeled gRNA resulted in a gradual downward shift in the Gag–RNA complex, leading to the reappearance of dimeric RNA (Fig. 6B). On the other hand, the *env* spliced RNAs were unable to effectively compete with the labeled gRNA for Pr50^Gag^ binding since dimeric RNA band could be detected only at 200 and 400 nM of competitor *env* mRNA (Fig. 6C; lanes 4–8). The reappearance of labeled dimeric RNA upon the addition of Proteinase K to the Gag-*env* mRNA complexes supports this assertion (Fig. 6C; lane 9).

Overall, these results suggest that in the case of FIV, the association and dissociation of Gag–RNA during electrophoretic migration is fast; hence, the formation of these complexes is dynamic. Nonetheless, a preferential binding of Pr50^Gag^ to the unspliced gRNA could be observed when compared to *env* spliced RNA.

### Filter-binding assays indicate that the U^85^CUG^88^ sequence is involved in Pr50^Gag^ binding

**FIGURE 7.**
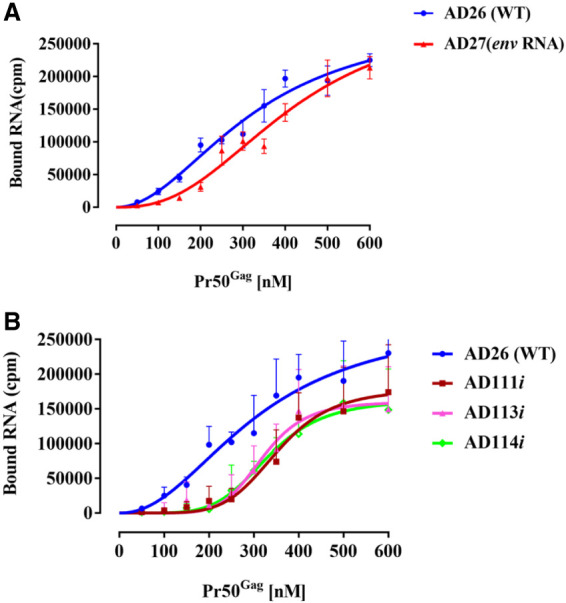
Binding of FIV unspliced gRNA, spliced *env* mRNA, and mutant gRNAs to FIV Pr50^Gag^ analyzed by filter-binding assay. The membrane-bound radioactivity of: (*A*) The WT AD26 gRNA and the spliced *env* mRNA, and (*B*) the WT AD26 gRNA and the selected U^85^CUG^88^ mutant RNAs. Note that mutations in U^85^CUG^88^ not only affected the RNA binding affinity, but also the binding plateau. Error bars represent the SD from the mean of three independent experiments.

Having established the role of the U^85^CUG^88^ sequence in FIV gRNA packaging and propagation (Fig. 3) and preferential binding of Pr50^Gag^ to gRNA over *env* spliced mRNA (Fig. 6), we used a noncompetitive experimental set up to determine whether Gag binds to U^85^CUG^88^. Toward this end, filter-binding assays were performed using radiolabeled WT gRNA (U^85^CUG^88^), spliced *env* mRNA, and selected U^85^CUG^88^ substitution mutants: AD111*i* (C^85^CCG^88^), AD113*i* (U^85^CCA^88^), and AD114*i* (U^85^CUA^88^) RNAs (Fig. 7A,B). As can be observed from Figure 7A, the *env* spliced mRNA (AD27) was less efficiently bound by Pr50^Gag^ than the WT unspliced gRNA (AD26), even though, the difference between the two RNAs was less pronounced than in the competitive set-up (Fig. 6B,C), as expected. Furthermore, all the U^85^CUG^88^ mutants tested consistently showed reduced levels of binding to Pr50^Gag^ compared to the WT (AD26) (Fig. 7B): not only the binding curves of the mutant RNAs were shifted toward higher P50^Gag^ concentrations, but in addition, the binding plateaus were lower, suggesting that these mutants have reduced binding affinity and binding stoichiometry compared to the WT gRNA. Reduced level of Pr50^Gag^ binding to these mutant RNAs corroborates well with reduced level of gRNA packaging and propagation obtained for these mutants (Fig. 3D,E). Taken together, these results confirm the unequivocal role of U^85^CUG^88^ in FIV gRNA packaging and Pr50^Gag^ binding. The U^85^CUG^88^ stretch may recruit P50^Gag^ either directly by functioning as a Gag binding site or indirectly via a yet-to-be-identified noncanonical tertiary interaction. Alternatively, mutations in the U^85^CUG^88^ sequence might indirectly affect Pr50^Gag^ binding by inducing an aberrant folding of the mutated RNAs.

In order to confirm the direct binding of Pr50^Gag^ to the U^85^CUG^88^ sequence, we attempted to observe the footprint of this protein on the WT unspliced gRNA; however, we were unable to detect any significant reactivity change within the first 500 nt of the gRNA in the presence of Pr50^Gag^ (data not shown). This observation is consistent with the very dynamic nature of the FIV gRNA/Pr50^Gag^ observed in our band-shift experiments (Fig. 6).

### The loop comprising the U^85^CUG^88^ stretch is base paired in the spliced *env* mRNA

**FIGURE 8.**
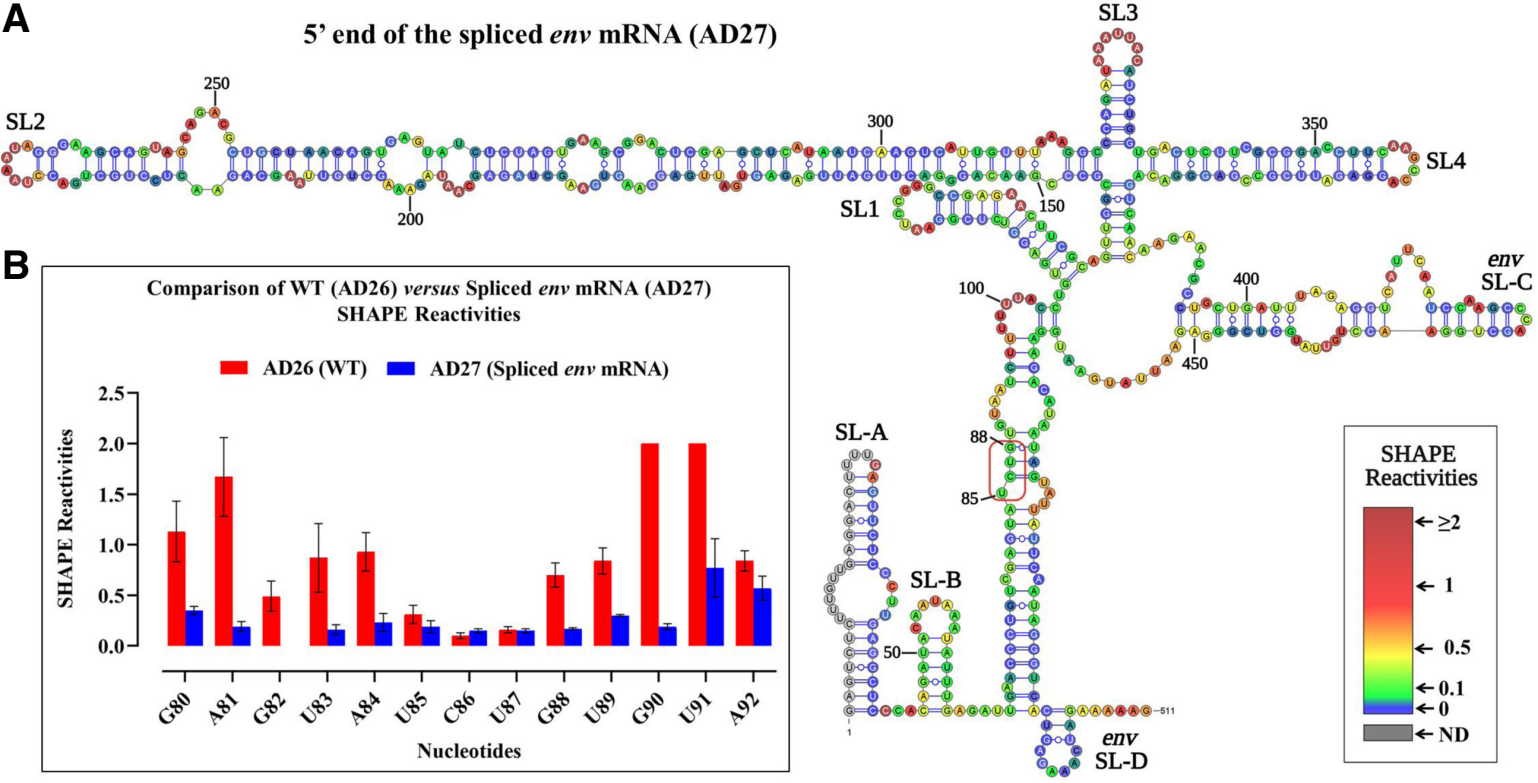
hSHAPE-validated secondary structure model of the first 744 nt of FIV spliced *env* mRNA (AD27). (*A*) The reactivity of each nucleotide in the RNA secondary structure is color-coded as indicated in the SHAPE reactivity key. The U^85^CUG^88^ motif is boxed in red, showing the base paired nature of the U^85^CUG^88^ stretch in the spliced *env* mRNA. (*B*) Histogram comparing the SHAPE reactivities of nucleotides 80–92 between the WT (AD26) and spliced *env* mRNA (AD27).

The U^85^CUG^88^ stretch is crucial for FIV gRNA packaging, but is positioned upstream of the mSD; therefore, it is part of both the unspliced gRNA (AD26) as well as the spliced *env* (AD27) mRNA. Despite this, our in vitro biochemical studies revealed a weaker affinity of spliced *env* mRNA (AD27) to Pr50^Gag^ when compared to the unspliced (AD26) RNA (Figs. 6 and 7). Such a reduced affinity could be explained if the U^85^CUG^88^ motif would not be accessible in the spliced *env* mRNA. To validate this hypothesis, hSHAPE was performed on the in vitro transcribed *env* spliced mRNA, and SHAPE reactivities were used as constraints to model the secondary structure of the *env* mRNA. The resulting secondary structure model of the *env* mRNA revealed that SL1–SL4 adopted the same secondary structure as in the unspliced RNA (Fig. 8A). However, the SHAPE reactivities of nucleotides 80–92 were significantly reduced in the spliced RNA as compared to the unspliced RNA (Fig. 8B). As a result, the large internal loop comprising the U^85^CUG^88^ stretch in the unspliced RNA now became part of two consecutive helices (Fig. 8A). Sequestration of the U^85^CUG^88^ stretch may abrogate its role in Pr50^Gag^ binding, thus potentially limiting selection and packaging of *env* spliced RNA.

### hSHAPE structural model of mutant RNAs

**FIGURE 9.**
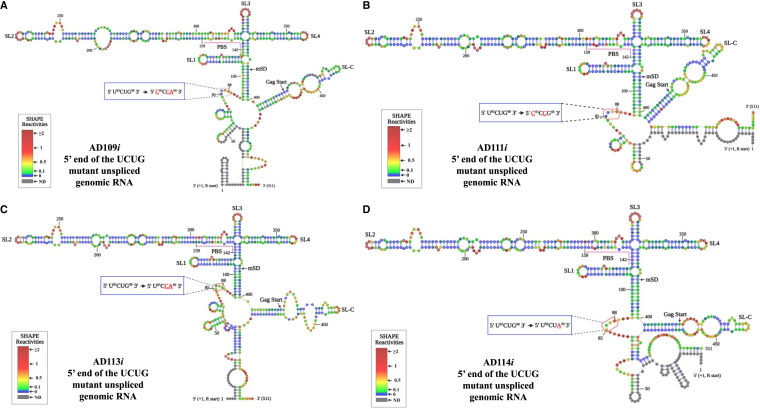
hSHAPE-validated secondary structure models of the first 744 nt of FIV gRNA harboring mutations in U^85^CUG^88^. (*A*) Mutant AD109*i*. (*B*) Mutant AD111*i*. (*C*) Mutant AD113*i*. (*D*) Mutant AD114*i.* The reactivity of each nucleotide in the RNA secondary structures is color-coded as indicated in the SHAPE reactivity key. The substitution mutations introduced in the U^85^CUG^88^ motif are highlighted and boxed in red and enlarged as insets. The hSHAPE reactivity data for each mutant is provided in Supplemental Table S2. (PBS) Primer binding site.

Our in vitro dimerization assays indicated that the dimerization ability of the U^85^CUG^88^ mutants was unaffected (Fig. 4E,F). On the other hand, gRNA packaging (Fig. 3) and in vitro Pr50^Gag^ binding of U^85^CUG^88^ mutant RNAs (Fig. 7) were significantly reduced suggesting that the structure of mutant RNAs could have been compromised. Thus, in order to check this possibility, hSHAPE experiments were conducted on mutants AD109*i*, AD111*i*, AD113*i*, and AD114*i* (Fig. 9A–D). Overall, the higher-order structures of these mutant RNAs were similar to that of the WT RNA and the substituted nucleotides remained unpaired, as in the case of WT gRNA (Fig. 9A–D). Furthermore, the reactivity of most of the nucleotides in stable regions, such as SL1, SL2, SL3, and SL4 remained unchanged. This suggests that the loss of Pr50^Gag^ binding to the mutant RNAs was not due to any global structural change in these RNAs, but directly due to substitutions introduced into the U^85^CUG^88^ motif, which may constitute a specific Pr50^Gag^ binding site. We observed that the predicted structural motifs are variable at the mutant RNA 5′ and 3′ ends, which could be due in part to the unavailability of reactivity data of nucleotides at either ends of the RNAs. Significantly, we also observed changes in the reactivities of several nucleotides in SL-C region that were specific to each substitution mutation introduced in the U^85^CUG^88^ stretch (Fig. 9A–D). These data suggest that tertiary interactions may establish between the loop containing the U^85^CUG^88^ sequence and the region located between nucleotides ∼410–460.

## DISCUSSION

Over the years, significant advances have been made in understanding the selective RNA packaging process for several lentiviruses, such as HIV-1, HIV-2, and SIV (D'Souza and Summers 2005; Lever 2007; Johnson and Telesnitsky 2010; Abd El-Wahab et al. 2014; Ali et al. 2016; Comas-Garcia et al. 2016; Mailler et al. 2016; Dubois et al. 2018; Pillai et al. 2021; Umunnakwe et al. 2021; Chameettachal et al. 2023). In this study, we aimed to better understand this complex process using FIV as a model system. In FIV, the RNA packaging determinant(s) are comprised of two discontinuous core regions that fold into a higher-order structure (Mustafa et al. 2005; Kenyon et al. 2008, 2011). However, the minimal RNA region that could be involved in interacting with the Gag during the FIV gRNA packaging process still remains unknown. Thus, the goal of this study was to delineate gRNA regions or structural elements that are crucial for packaging and Gag binding. Using a systematic mutational approach, we examined the FIV packaging signal RNA to locate the sequences that are important in selective packaging by the Gag polyprotein.

Previous studies have demonstrated the role of purine-rich regions overlapping with the DIS or adjacent to it as binding sites for retroviral Gag protein facilitating efficient gRNA packaging (Paillart et al. 1996, 1997; Zeffman et al. 2000; Lever 2009; Moore and Hu 2009; Moore et al. 2009; Didierlaurent et al. 2011; Bell and Lever 2013; Nikolaitchik et al. 2013; Abd El-Wahab et al. 2014; Smyth et al. 2015; Bernacchi et al. 2017; Mustafa et al. 2018; Nikolaitchik et al. 2020; Chameettachal et al. 2021; Pillai et al. 2021; Pitchai et al. 2021; Umunnakwe et al. 2021). This prompted us to investigate the purine-rich regions in close proximity to the previously reported DIS (Kenyon et al. 2011) in the FIV packaging signal RNA. Surprisingly, none of these mutations (AD101–AD106; Fig. 1) had any effect on RNA packaging and virus propagation, even in the region encompassing the DIS (AD104 and AD105; Fig. 1), indicating that there may be other sequences in addition to the previously proposed DIS (Kenyon et al. 2011) that mediate intermolecular interactions between the two RNAs molecules during FIV gRNA dimerization and packaging.

Next, we examined the role of sequences in SL1 in FIV gRNA packaging since it was one of the unexplored structural motifs present in the region that has been shown to be indispensable during the FIV gRNA packaging process (Mustafa et al. 2005; Kenyon et al. 2008, 2011). The substitution mutation introduced in the loop of SL1 (AD107: Fig. 2D,E) while maintaining the size of the loop did not show any detrimental effect on RNA packaging and propagation. On the other hand, a deletion of the entire SL1 (AD108; Fig. 2D,E) showed a more drastic effect on RNA packaging and propagation compared to the WT. It is conceivable that SL1 deletion affected the overall structure as it is found in a very central nodal area where several stem–loops (SL2, 3, 4) meet, suggesting that the effect of SL1 deletion on FIV gRNA packaging is more due to an indirect effect in this case. It is also possible that due to its close proximity with the PBS, the SL1 deletion may have had structural effects on the PBS domain, thus affecting reverse transcription. This is consistent with efficient RNA packaging of mutant AD108 in the virus particles compared to RNA propagation, which is dependent on successful reverse transcription and integration of the reverse transcribed RNA (Fig. 2D,E). Similar effects have been earlier reported in viruses harboring mutations in the close vicinity of the PBS (Mikkelsen et al. 1996; Beerens et al. 2001; Ooms et al. 2007; Oh et al. 2008; Aktar et al. 2014; Chameettachal et al. 2021; Song et al. 2021).

Considering the limited impact of mutations on purine-rich regions and SL1, we investigated other potential regions in the FIV packaging signal RNA that could be involved in the selective packaging of FIV gRNA. The stretch U^85^CUG^88^, which base pairs with the downstream sequences U^413^GGG^416^ immediately after an LRI in an earlier proposed secondary structure model (Kenyon et al. 2008, 2011), attracted our attention. Interestingly, mutational analysis of U^85^CUG^88^ revealed that all the 4 nt are crucial for FIV gRNA packaging and propagation (Fig. 3D,E), suggesting that this sequence may constitute a Pr50^Gag^ binding site. It is worth mentioning that this U^85^CUG^88^ motif falls within a region that has earlier been shown to be indispensable for FIV gRNA packaging (Mustafa et al. 2005).

To our surprise, mutations in the proposed base pairing partner U^413^GGG^416^ did not show any effect on RNA packaging and propagation (Fig. 3), indicating that the U^413^GGG^416^ sequence is not involved in FIV gRNA packaging, and suggesting that this sequence may not be base paired with U^85^CUG^88^, as initially proposed (Kenyon et al. 2011). The structural model presented in our study using hSHAPE confirmed that in fact the U^85^CUG^88^ motif is present in an unpaired fashion as a part of a large internal loop (Fig. 5).

In vitro RNA–protein binding experiments further confirmed that all 4 nt in the U^85^CUG^88^ stretch contribute to Pr50^Gag^ binding, and thus could act as a potential Pr50^Gag^ binding site during the FIV gRNA packaging process (Figs. 6 and 7). This scenario would somewhat be similar to MoMuLV where it has been shown that a UCUG motif (that is obscured in the monomeric RNA and becomes exposed upon gRNA dimerization) is important for high affinity binding to NC as well as to full-length Gag (D'Souza and Summers 2004; Gherghe et al. 2010; Miyazaki et al. 2010). However, unlike MoMuLV, where four UCUG stretches are required for efficient gRNA packaging, we identified only a single unpaired U^85^CUG^88^ motif acting as a potential Gag binding site. In this respect, the FIV Pr50^Gag^ binding to the U^85^CUG^88^ motif is more similar to HIV-1 where the specific Gag binding motif is unpaired in the monomeric conformation (Abd El-Wahab et al. 2014). Having said this, we cannot rule out that other UCUG-like sequences or unpaired Gs could bind to Gag since unpaired Gs have been shown to contribute to HIV-1 RNA packaging (Comas-Garcia et al. 2018; Nikolaitchik et al. 2020). This is because many of these nucleotides may act synergistically, and mutating a single or a short stretch of unpaired Gs may not significantly affect RNA packaging. Furthermore, in the case of MPMV, it has been demonstrated that two stretches of purines act redundantly in RNA packaging (Ali et al. 2020; Pitchai et al. 2021). Consequently, mutating one motif may not lead to a loss of packaging due to compensation by the other region. Thus, if such redundant regions exist in FIV, it would be difficult to identify them using the current experimental approach. Given the fact that FIV uses a single U^85^CUG^88^ motif to bind to Gag to augment gRNA packaging, it is reasonable to suggest that the mechanism(s) that regulates dimeric RNA packaging in MoMuLV is different from the one that regulates HIV-1 or FIV gRNA packaging.

In an attempt to establish a structure–function relationship during FIV gRNA packaging, we performed hSHAPE on selected U^85^CUG^88^ substitution mutants. Comparing the biological gRNA packaging data to the structural models for different mutants revealed an interesting phenomenon. All substitution mutants that were investigated, despite having severe effects on RNA packaging, maintained the overall RNA secondary structure (except for variabilities in reactivities toward the 5′–3′ ends), especially the large loop harboring the U^85^CUG^88^ or its mutant forms (Fig. 9A–D). These data strongly suggest that the effect of mutations in the U^85^CUG^88^ motif on Pr50^Gag^ binding (Fig. 7), RNA packaging (Fig. 3), and viral propagation (Fig. 3) are direct effects, rather than indirect effects due to global refolding of the mutant RNAs. These data suggest that the U^85^CUG^88^ motif is an authentic Pr50^Gag^ binding site. However, the U^85^CUG^88^ motif may also recruit Gag and allow gRNA packaging by establishing noncanonical tertiary interactions with the region located between nucleotides ∼410–460, as suggested by the observation that mutations in U^85^CUG^88^ resulted in subtle hSHAPE reactivity changes in the latter region (Figs. 5 and 9).

The phenomenon of maintaining the overall RNA secondary structure of U^85^CUG^88^ substitution mutants, especially the large loop harboring the U^85^CUG^88^ motif is reminiscent to what has been observed in the case of MMTV. In this case, the Gag binds to single-stranded purines (ssPurines) located in the apical loop of the bifurcated stem–loop SL4 (Aktar et al. 2014; Mustafa et al. 2018; Chameettachal et al. 2021). Interestingly, MMTV gRNA packaging was lost in ssPurines substitution mutants despite having proper folding of SL4 with an apical loop similar to the WT but with a mutated sequence (Mustafa et al. 2018; Chameettachal et al. 2021). Furthermore, similar to MMTV, the substitution mutants in the 5′ U^85^CUG^88^ 3′ also dimerized like WT gRNA (Fig. 4), but their ability to package was severely compromised, reinforcing the crucial role of the 4 nt in the 5′ U^85^CUG^88^ 3′ stretch during FIV gRNA packaging.

This raises the question of what prevents the specific encapsidation of host RNAs into viral particles since such small RNA motifs most likely are present in cellular RNAs as well. It is now becoming apparent that short purine-rich RNA sequences can drive RNA packaging among retroviruses by acting as sites of Gag “nucleation.” For instance, a stretch of purines on MMTV gRNA or multiple guanosines spread across the minimal HIV-1 packaging signal RNA are critical for RNA packaging (Nikolaitchik et al. 2020; Chameettachal et al. 2021). It is well established that retroviruses can encapsidate both noncoding host RNAs and mRNAs (Rulli et al. 2007; Mustafa et al. 2012). However, Gag binding to cellular RNAs may be less efficient than viral RNAs, as has been shown for the cellular 7SL RNA packaged into HIV-1 virions (Keene and Telesnitsky 2012). Thus, the mere presence of these short sequences is not sufficient for selective RNA encapsidation and the RNA structure within which they are found is equally crucial (e.g., the unpaired status of these nucleotides which is critical for Gag binding). Furthermore, their flanking regions may also play a vital role in this process since in HIV-1, other negative or positive regulatory elements within the RNA can also affect this process (Abd El-Wahab et al. 2014).

### Conclusions

Taken together, our study demonstrates that the U^85^CUG^88^ motif is critical for the packaging of FIV gRNA and Pr50^Gag^ binding. The fact that FIV uses U^85^CUG^88^ as a potential Gag binding site to facilitate gRNA packaging would be similar to MoMuLV (D'Souza and Summers 2004; Gherghe et al. 2010; Miyazaki et al. 2010) when compared to MMTV, MPMV, or lentiviruses, such as HIV-1, HIV-2, and SIV, which use purine-rich sequences for this purpose (Baig et al. 2009; Abd El-Wahab et al. 2014; Keane et al. 2015; Bernacchi et al. 2017; Nikolaitchik et al. 2020; Chameettachal et al. 2021; Pillai et al. 2021; Pitchai et al. 2021; Umunnakwe et al. 2021). Remarkably, we observed that FIV Gag binding is more dynamic than what has been observed in other retroviruses. Whether highly dynamic Pr50^Gag^ binding to the FIV RNA ψ is needed for gRNA packaging will require further investigations. Additional studies will also be required to identify tertiary interactions in the ψ region that may affect this process in FIV replication.

## MATERIALS AND METHODS

### FIV strain

The FIV Petaluma strain 34TF10 (GenBank accession number M25381.1) was used for the construction of vectors used in this study (Talbott et al. 1989).

### Cloning and site-directed mutagenesis

Splice overlap extension (SOE) polymerase chain reaction (PCR) was used to introduce the desired mutations into the FIV ψ RNA using the subgenomic transfer vector TR394 (Browning et al. 2001) as the template. OTR 249 and OTR 684 were used as the outer sense (OS) and antisense (AS) primers, respectively. Sequences and details of all the primers used for cloning are provided in Supplemental Table S1. Inner primers containing the nucleotide changes to be introduced into the template (TR394) were used to incorporate the desired mutations. For each mutation, custom inner primers were designed and used along with OS (OTR 249) and AS primer (OTR 684) to perform the two first rounds of PCRs. Products from these PCRs were annealed and used as templates to conduct the second round of PCR using the OS and AS primers (OTR 249 and OTR 684). The resulting PCR product was digested with SpeI and BamHI and ligated to TR394 digested with the same restriction enzymes. For in vitro transcription of FIV packaging sequences, the subgenomic transfer vector TR394 or its mutant clones were used as templates to amplify the 5′ end of the FIV gRNA corresponding to nucleotides 1–744 (nucleotide 1 corresponds to the start of the R [repeat] region). Primers used for amplification included a forward primer containing the T7 promoter sequence and a HindIII restriction site (OTR 1616) and a reverse primer containing an XmaI restriction site (OTR 1360) (Supplemental Table S1). The final amplified products were cleaved by HindIII and XmaI and cloned into pIC19R (Marsh et al. 1984), a pUC-based cloning vector. Using a similar strategy and keeping the same viral sequence length (744 nt), we also created an in vitro transcription plasmid containing the 5′ end region of the FIV *envelope (env)* spliced mRNA.

### In cell genetic complementation assay/virus production, and target cell transduction

The gRNA packaging and propagation efficiencies of mutants were investigated using a previously established in cell genetic complementation assay (Supplemental Fig. S1; Browning et al. 2001). This assay uses three expression plasmids: MB22 (FIV Gag/Pol expression plasmid) (Browning et al. 2001), MD.G (vesicular stomatitis virus glycoprotein [VSV-G] expression plasmid) (Naldini et al. 1996), and FIV subgenomic transfer vector TR394 (Browning et al. 2001). TR394 contains the *cis*-acting sequences vital for RNA packaging, reverse transcription, and integration, and also expresses the *hygromycin B phosphotransferase* gene from the simian virus 40 (SV40) promoter as a marker to assess successful transduction (transfer or propagation of the packaged RNA) into target cells. A fourth plasmid DNA (pSEAP2-control vector, Clontech) expressing the *secreted alkaline phosphatase* (*SEAP*) gene was used to determine the transfection efficiency. The four plasmids were cotransfected into the virus-producing human embryonic kidney cells (HEK293T) using a commercially available calcium phosphate transfection kit (Thermo Fisher Scientific) in accordance with the manufacturer's instructions. The pseudotyped virus particles thus generated were purified as previously described (Browning et al. 2001), and the viral RNA was extracted using TRIzol (Thermo Fisher Scientific), as per the manufacturer's instructions. A portion of the virus stock was used to infect HeLaT4 target cells in the presence of 1 µg/mL diethylaminoethyl (DEAE)-dextran, a polycation polymer to increase virus uptake by the cells. Selection of target cells and scoring of Hyg^R^ colonies were performed as described previously (Browning et al. 2001). The viral titers were normalized to the transfection efficiency of each transfected culture assessed by the relative SEAP values observed. The number of Hyg^R^ colonies obtained should be directly proportional to the amount of RNA packaged into the virus particles, providing an indirect estimate of RPE.

### Nucleocytoplasmic fractionation, isolation of RNA, and cDNA preparation

Cellular nucleocytoplasmic fractionation was performed as described earlier (Akhlaq et al. 2018). The cytoplasmic and viral RNAs were treated with DNase (Turbo DNase, Thermo Fisher Scientific) and tested for the presence of DNA using FIV-specific primers (OTR 660 and OTR 662, Supplemental Table S1) to ensure that the RNA preparations were not contaminated with any residual plasmid DNA from the process of transfection. Next, the DNased-RNAs were reverse transcribed using random hexamers (5′ NNNNNN 3′) and M-MLV reverse transcriptase (Promega), as previously described (Ali et al. 2020; Chameettachal et al. 2021; Pillai et al. 2021). In order to ensure proper fractionation and the quality of the cDNAs prepared, multiplex RT-PCR was performed using unspliced β-actin primers (OTR 582; S and OTR 581; AS) (Tan et al. 1995; Supplemental Table S1) as well as primers for 18S rRNA (18S Quantum competimer control, Ambion), as described previously (Mustafa et al. 2005, 2012; Ghazawi et al. 2006; Rizvi et al. 2010).

### Quantitative real-time PCR

The subgenomic transfer vector RNA expression in the cytoplasm and the packaged RNA in the pelleted virus particles from the transfected cultures were measured by quantitative real-time PCR (qRT-PCR) to estimate the RPE of each mutant. Toward this end, we quantified the transfer vector RNA expressed in the cytoplasm and packaged RNA in the pelleted viral particles using an in-house SYBR Green-based qRT-PCR assay, as described previously (Krishnan et al. 2019; Pillai et al. 2021), using primers OTR 1648 and OTR 1649 (Supplemental Table S1) targeting a region within the U5/UTR region of the FIV genome. Spliced β-actin was used as an internal control to normalize the relative expression of cytoplasmic and packaged viral RNAs (Krishnan et al. 2019; Pillai et al. 2021). qRT-PCR was carried out using an Applied Biosystems 7500 ABI QuantStudioTM7 Flex System (Applied Biosystems) in triplicate for 40 cycles with an annealing temperature of 60°C. Cytoplasmic expression of each mutant was determined by comparing the relative quantification (RQ) values for each mutant RNA compared to the WT (TR394) transfer vector RNA. Finally, to determine the packaging efficiency of each vector RNA, the ratio of the viral RQ to the corresponding cytoplasmic RQ for each sample was determined and the values represented relative to the WT (TR394).

### In vitro RNA transcription and purification

For in vitro RNA transcription, appropriate plasmids were linearized using SmaI restriction enzyme, and the linearized DNA was extracted with RotiAqua (Carl Roth, Germany), as per manufacturer's instructions. Extracted DNA was resuspended in 30 µL Milli-Q water and 20–30 µg of DNA was used as a template for in vitro transcription. A 300 µL transcription reaction mixture containing 10× T7 buffer (400 mM Tris-HCl pH 8.0 at 37°C; 150 mM MgCl_2_; 500 mM NaCl), 500 mM DTT, 10 mg/mL BSA, 40 U/µL RNasin, 100 mM of each NTP, 100 mM spermidine, 0.5% Triton X-100, 0.2 U/µL pyrophosphatase, template DNA, and T7 RNA polymerase was incubated at 37°C for 3 h, treated with 10 U/µL DNase I, and extracted using the phenol–chloroform method. Purification and quality control of the in vitro transcribed RNA by gel filtration chromatography was performed as described (Chameettachal et al. 2021). Internally radiolabeled RNAs were obtained as previously described (Chameettachal et al. 2021) and purified using Micro Bio-Spin chromatography columns according to manufacturer's instructions.

### In vitro dimerization assay

Genomic RNA dimerization was tested essentially as described previously (Chameettachal et al. 2021), except that the monomer and dimer buffers contained 30 mM HEPES pH 8 buffer, instead of 30 mM Tris pH 7.5, and 40 mM or 300 mM KCl, 0.1 mM or 5 mM MgCl_2_ to obtain RNA monomers or dimers, respectively. After native agarose gel electrophoresis, gels were scanned by the Gel Doc EZ Imager (BioRad) and peak areas, corresponding to the monomeric and dimeric forms, were quantified using Image Lab (BioRad) software, as described previously (Chameettachal et al. 2021).

### High-throughput selective 2′-hydroxyl acylation analyzed by primer extension

For hSHAPE modification, 2 pmol of RNA in 8 µL of Milli-Q water was denatured at 90°C and chilled on ice for 2 min. RNA was folded by the addition of 2 µL of 1× dimer buffer (30 mM HEPES, pH 8, 300 mM KCl, 5 mM MgCl_2_) followed by incubation for 20 min at 37°C. To each sample, 2 µg of total yeast tRNA (Sigma-Aldrich) in 1× dimer buffer was added, and the mixture was incubated for 10 min at room temperature. This was followed by treating the reaction mixture with 3 µL of a 300 mM BzCN solution in anhydrous DMSO for 1 min at room temperature to modify the RNA, and the reaction was stopped by adding 82 µL water. The negative control samples were treated in a similar fashion but using only DMSO without any BzCN. All samples were precipitated by the addition of three volumes of cold ethanol (−20°C), 1 µg of glycogen, and 1/10 volume of 3 M sodium acetate (pH 5) for 30 min in a dry ice/ethanol bath. The resulting precipitates were collected by centrifugation at 13,000 revolutions per minute (rpm) for 30 min at 4°C using a tabletop microfuge. The RNA pellets were washed twice with cold 80% ethanol, air dried, and resuspended in 7 µL of Milli-Q water.

### cDNA synthesis and capillary electrophoresis

RNAs modified with BzCN as well as the negative control (unmodified RNAs) were subjected to reverse transcription in order to identify the modifications as described in Chameettachal et al. (2021) and Gilmer et al. (2022). Three sets of VIC-labeled primers (AS_FIV_1, AS_FIV_3, AS_FIV_5; Supplemental Table S1) were used to reverse transcribe the RNA samples. Briefly, 1 µM of the VIC-labeled primers were denatured and used for cDNA preparation using avian myeloblastosis virus (AMV) RT. For sequencing reactions, reverse transcription was performed using three sets of 2 µM NED-labeled primers (AS_FIV_2, AS_FIV_4, AS_FIV_6; Supplemental Table S1) as described previously (Chameettachal et al. 2021; Gilmer et al. 2022). The cDNAs thus generated from both reactions were combined, precipitated, and denatured prior to their analysis, using the Applied Biosystems 3130xl Genetic Analyzer. The electropherograms thus obtained were analyzed with QuShape software (Karabiber et al. 2013). Normalized SHAPE reactivity data from at least three to four independent experiments were used as pseudoenergy constraints to fold the RNA secondary structure of the FIV packaging signal using the program RNAstructure, version 6.1 (Reuter and Mathews 2010). RNA structures were then drawn using VARNAv3-93 and hSHAPE reactivity was incorporated, as described previously (Darty et al. 2009).

### Purification of FIV Pr50^Gag^ and western blot analysis

In order to purify FIV Pr50^Gag^-His_6_-tagged protein, bacterial cultures were suboptimally expressed and the protein was purified by immobilized metal affinity chromatography (IMAC), and further characterized by western blotting, as described previously (Chameettachal et al. 2018; Pitchai et al. 2018; Krishnan et al. 2019).

### Dynamic light scattering

The bacterially expressed and purified Pr50^Gag^ polyprotein was characterized by DLS in HEPES-KOH buffer (pH 8) as described earlier (Chameettachal et al. 2021). By considering the proteins in solution as spheres, the diffusion coefficients (*D*) were used to determine the hydrodynamic radius (Rh) of the molecules through the application of the Stokes–Einstein equation:

$$D=\frac{kT}{6\pi \mu Rh},$$

in which *k* is the Boltzmann constant, *T* is the temperature, and *μ* is the viscosity of the solvent.

### Band-shift and band-shift competition assays

Protein–RNA binding was carried out under increasing concentrations of purified FIV Pr50^Gag^ and analyzed as described (Chameettachal et al. 2021) with the following modifications: 25,000 counts per minute (cpm) of internally ^32^P-labeled RNA was used (instead of 50,000 cpm) and all buffers contained HEPES pH 8 instead of Tris pH 7.5. For competition experiments, increasing concentrations of unlabeled competitor RNAs (up to 400 nM) were added to the reaction mixture, and binding was performed with a constant concentration of FIV Pr50^Gag^ (400 nM).

### Filter-binding assays

The standard filter-binding assay was performed using 9 × 12 centimeter (cm), 0.45 µm nitrocellulose membrane (BioRad). Ten thousand cpm of ^32^P-labeled RNA, 5 nM of the cognate unlabeled RNA, and 0.4 µg of yeast tRNA were denatured for 2 min at 90°C and chilled on ice for 2 min. The denatured RNAs were then incubated for 60 min at 37°C in 1× RNA folding buffer (30 mM Tris-HCl pH 8, 300 mM NaCl, 5 mM MgCl_2_), 5 U of RNasin, and 0.01% Triton X-100. Reaction mixtures were incubated for another 30 min with increasing concentrations of protein in 1× protein buffer (30 mM Tris-HCl pH 8, 300 mM NaCl, 5 mM MgCl_2_, 10 mM DTT, 0.02 mg/mL BSA) to form the protein–RNA complexes. The protein–RNA complexes were further stabilized by incubating the samples on ice for 30 min. Nitrocellulose membranes were prewetted with 1× TBS (Tris-buffered saline: 20 mM Tris-HCl pH 7.5 and 500 mM NaCl) for 10 min at room temperature. After gently drying the membrane on a filter paper, it was carefully positioned onto the Bio-Dot Microfiltration Apparatus connected to a suction drainage. Each well was prewashed with 100 µL of 1× TBS before the application of protein–RNA mix. After adding 40 µL of cold 1× Gag binding buffer (30 mM Tris-HCl pH 8, 300 mM NaCl, 5 mM MgCl_2_), 20 µL of reaction mixture was added to each well and incubated at room temperature for 10 min. The nitrocellulose membrane was washed three times with 100 µL of cold 1× Gag binding buffer to remove the unbound proteins or RNA and air dried after the final wash. The filters were quantified using ImageQuant (Cytiva) software after being exposed with an Imaging Plate (Fujifilm) and scanned with an FLA 5000 (Fuji) scanner (Abd El-Wahab et al. 2014).

### Statistical analysis

Individual data set points for relative RNA packaging and RNA propagation efficiencies were plotted using GraphPad Prism v8 software. Statistically significant differences between the mutant and the WT clones were estimated by the paired, two-tailed, Student's *t*-test. *P*-values <0.05 were considered significant and denoted by one to three stars, depending upon the values obtained ([*] 0.01 ≤ *P* < 0.05, [**] 0.001 ≤ *P* < 0.01, [***] 0.0001 ≤ *P* < 0.001).

## SUPPLEMENTAL MATERIAL

Supplemental material is available for this article.

## Figures and Tables

**Anjana Krishnan:**
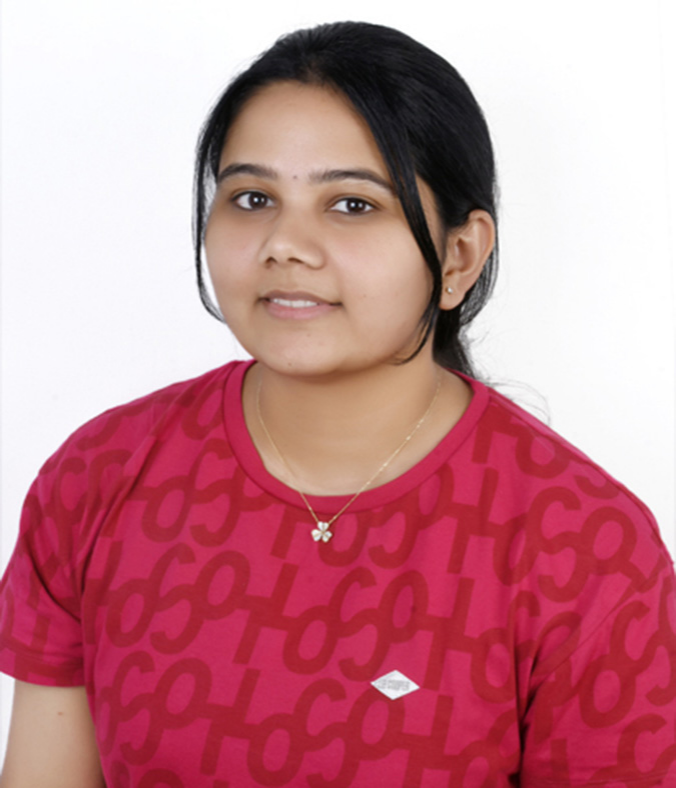


## MEET THE FIRST AUTHOR

***Meet the First Author(s)* is an editorial feature within *RNA*, in which the first author(s) of research-based papers in each issue have the opportunity to introduce themselves and their work to readers of *RNA* and the RNA research community. Anjana Krishnan is the first author of this paper, “Identification of a putative Gag binding site critical for feline immunodeficiency virus genomic RNA packaging.” Anjana is currently a PhD candidate with Dr. Tahir A. Rizvi at the College of Medicine and Health Sciences, United Arab Emirates University. The main focus of Anjana's research is to understand the genomic RNA (gRNA) encapsidation of feline immunodeficiency virus (FIV) during the process of its assembly into virion, and to identify the Gag binding site(s) on the packaging signal RNA.**

**What are the major results described in your paper and how do they impact this branch of the field?**

This study essentially identified a stretch of 4 nt, UCUG, within FIV RNA packaging signal (psi) that acts as a potential Gag binding site. These sequences are observed in an unpaired manner in the unspliced, full-length gRNA, an aspect which is critical for binding to Gag and its selective packaging into assembling viral particles. Base pairing of this sequence motif (UCUG) in the spliced Env mRNA limits its ability to bind to Gag, thus excluding its encapsidation into the newly assembling virions. These findings should contribute to our understanding how retroviruses in general, and FIV in particular, have evolved mechanism(s) to selectively package gRNA while excluding Env spliced and other cellular RNAs. Being a nonprimate lentivirus, FIV-based vectors are a good candidate for delivering therapeutic genes during human gene therapy, therefore, identifying the minimal sequences required for FIV gRNA packaging should facilitate improving the design and safety of such FIV-based vectors.

**What led you to study RNA or this aspect of RNA science?**

Our laboratory is interested in studying retroviral replication and specifically how retroviruses recognize and capture their genomes for packaging into newly assembling virions. Since retroviruses contain single-stranded RNA and not DNA as their genome, this was the primary reason to study retroviral RNA genomes. While the field of RNA research is rapidly evolving, a lot remains to be discovered in this field, especially how the structural motifs within RNA control its function, especially in retroviruses. As it turns out, the secondary structure of retroviral RNAs not only regulates gene expression posttranscriptionally (for e.g., the Rev-responsive element of HIV), but also encapsidation (as observed in this and many other studies). Therefore, the study of RNA and how retroviral and/or cellular proteins interact with viral gRNA is a crucial aspect to study in the field of retrovirology.

**During the course of these experiments, were there any surprising results or particular difficulties that altered your thinking and subsequent focus?**

Yes, indeed we did encounter several surprises during the course of our study. For example, we observed important differences in the secondary RNA structure of FIV psi from what has been published earlier, where the proposed biochemically validated structure was not verified using biologically relevant assays. In this study, we were able to correlate the presence of these differences with results from a biological assay, leading to a newly validated secondary structure of FIV psi. We also observed that the RNA structure of FIV psi was dynamic in nature and that may partly explain the differences between the two studies. The dynamic nature of FIV Gag–RNA binding made it a challenge to perform more accurate in vitro binding experiments. Such a dynamic interaction has not been previously observed with other retrovirus psi in which Gag binding site(s) have been identified, such as human immunodeficiency virus (HIV-1), mouse mammary tumor virus (MMTV), Mason–Pfizer monkey virus (MPMV), and murine leukemia virus (MLV).

**What are some of the landmark moments that provoked your interest in science or your development as a scientist?**

The moment that provoked my interest in science started very early on and perhaps from attending “science exhibitions” that were conducted at my school every year. For these exhibitions, students were asked to craft their ideas for displaying to the public. It was really interesting to see the creativity and innovative thoughts presented during these science exhibitions which could be transformed into reality; thus igniting my interest in science and research.

**If you were able to give one piece of advice to your younger self, what would that be?**

I would like to tell my younger self that curiosity, patience, and perseverance are the major driving forces to stay active in today's science. Whenever faced with difficulties, it is important to take a step back and critically analyze the question that is being addressed as well as the data generated rather than giving up in haste, since the data, whether negative or positive, are directing us in some direction.
